# Perceived empathy and competence across psychotherapist appearance types: a quasi-experimental repeated-measures study

**DOI:** 10.3389/fpsyt.2026.1896992

**Published:** 2026-08-13

**Authors:** Teresa Festl-Wietek, Johannes Le Guellec, Rebecca Erschens, Tobias Albrecht, Bernd Herrmann, Stephan Zipfel, Anne Herrmann-Werner

**Affiliations:** 1 TIME - Tübingen Institute for Medical Education, University of Tübingen, Tübingen, Germany; 2 Department of Psychosomatic Medicine and Psychotherapy, University Hospital Tübingen, Tübingen, Germany; 3 Department of Otorhinolaryngology, Head and Neck Surgery, Medical Center - University of Tuebingen, Tübingen, Germany; 4 German Center for Mental Health (DZPG), Partner Site Tübingen, Tübingen, Germany

**Keywords:** appearance-based bias, competence, empathy, medical education, psychotherapy

## Abstract

**Introduction:**

Physical appearance, including body weight and clothing, can affect perceptions of physicians’ empathy and competence, thereby shaping the quality of the patient–physician relationship. Understanding these influences is essential for teaching about implicit bias in medical education. To examine how medical students’ evaluations of psychotherapists’ empathy and competence are influenced by external characteristics such as body weight and clothing style.

**Methods:**

The survey is a quasi-experimental, video-based repeated-measures design. A total of 330 second-year medical students participated. Videos of patient-psychotherapist communication were shown portraying four different types of psychotherapists: the white coat, Freud, ecological, and overweight types. In a second video the same scenarios were shown but the psychotherapist wore normal everyday clothing. Students rated psychotherapists shown in standardized visual stimuli using validated questionnaires, including the Jefferson Scale of Patient’s Perceptions of Physician Empathy, the Physician Compassion Questionnaire, and Osgood’s Semantic Differential Scale.

**Results:**

Of 355 eligible students, 330 participated (93.0%; mean age 23.0 years, SD = 3.35; 65.2% women). Therapist appearance systematically shaped students’ evaluations of empathy, compassion, prejudice, and competence. The overweight (OSS) type received the most favorable ratings overall, including for *taking the patient’s perspective* (M = 5.51, SD = 1.07), *inquiring about everyday life* (M = 5.88, SD = 1.21), and *understanding* (M = 5.51, SD = 1.14), whereas the Freud type was rated least favorably (e.g., *understanding*: M = 4.37, SD = 1.66). Significant type effects were observed for all empathy items (all p <.001). Students agreed that therapist appearance influenced their judgments, although differences between therapist types were not significant (p = .167).

**Conclusion:**

External characteristics significantly affected medical students’ evaluations of psychotherapists’ empathy and competence. These findings highlight the need to integrate reflection on physical appearance and bias into medical training to promote more objective and equitable patient care.

## 1 Introduction

A trusting physician–patient relationship is fundamental to effective medical care and is often shaped within the first moments of an encounter (1–5). Early judgments draw on verbal and nonverbal communication, but also on readily visible cues such as attire and grooming (2–4). While these cues may appear peripheral to clinical quality, they can meaningfully influence perceived professionalism and the willingness to engage in care (6). One mechanism frequently cited in this context is the *halo effect*, a systematic bias in which a salient characteristic disproportionately shapes global impressions, even when unrelated to the trait being judged (7). For example, individuals perceived as physically attractive may be judged as more competent (7, 8).

Within medical settings, two appearance-related cues have received particular attention: the physician’s white coat and body weight (4, 9–13). The white coat - despite increasing criticism for symbolizing traditional medical authority (14, 15) - continues to communicate professionalism, competence, trustworthiness, and scientific credibility to many patients (4, 9, 10, 14). At the same time, it may also reinforce distance and hinder patient-centered communication in some contexts (10, 16). In contrast, obesity is linked to pervasive negative stereotypes, and people with obesity are frequently stigmatized and socially disadvantaged worldwide (17–21). Although the prevalence of obesity among physicians appears comparable to that in the general population (12, 22, 23), physicians with overweight report professional disadvantages, including reduced perceived credibility, trustworthiness, and authority (11).

Beyond medicine, appearance-based expectations have also been described in psychotherapy. Von Sydow et al. reported that when students described “typical” psychotherapists, they frequently referred to a Freud-like prototype (24). In addition, they identified an “ecological” type characterized by an alternative lifestyle and attire (24). These findings suggest that professional prototypes may shape judgments in psychotherapy-related disciplines as well.

Taken together, existing literature indicates that visible characteristics—particularly clothing and body weight—can influence how clinicians are perceived (4, 9–14, 16, 17, 22, 23). Medical students in particular need to recognize early on in their studies that external characteristics can influence the patient-physician relationship and thus the adherence and treatment. They should learn to reflect on a meta-level and not allow themselves to be influenced by unconscious biases. However, literature is missing where medical students were confronted with visible characteristics of clinicians in fields where empathy, relational qualities and nonverbal communication are especially central, such as psychosomatic medicine. Addressing this gap is relevant not only for patient care but also for medical education, where recognizing and managing appearance-based bias is a key competency. Because clinicians working in psychosomatic medicine and psychotherapy typically do not wear the traditional white coat commonly associated with other medical specialties, this setting offers a particularly relevant context in which to examine how alternative aspects of professional appearance influence perceptions of therapists.

Therefore, this study examined whether and how medical students’ evaluations of psychotherapists vary as a function of external appearance. In a longitudinal observational design, we assessed perceived competence and empathy for psychotherapists portrayed with four distinct external characteristics: the white coat type, the Freud type, the ecological type and the overweight (OSS) type. Specifically, we addressed the following questions: 1) How do ratings of perceived competence and empathy differ across these appearance types? 2) To what extent do students report that visual appearance influenced their evaluations?

## 2 Materials and methods

### 2.1 Study design

This study employed a quasi-experimental, video-based repeated-measures design to examine whether external characteristics influence medical students’ evaluations of psychotherapists’ perceived competence and empathy during the winter term of the 2022–2023 at University of Tübingen. Students were invited to take part in the study during their communication or psychosomatic course respectively. Participants were eligible for inclusion if they were enrolled in a medical degree programme, were at least 18 years of age, and had sufficient proficiency in German to understand the study information and complete the study procedures. Individuals who did not meet any of these criteria were excluded.

### 2.2 Ethics

The study was approved by the Ethics Committee of the Tübingen Medical Faculty (No. 703/2022BO2). Participation was voluntary. All participants gave their written consent to participate. All data and responses were processed anonymously.

### 2.3 External characteristics of psychotherapists

**Figure 1:**
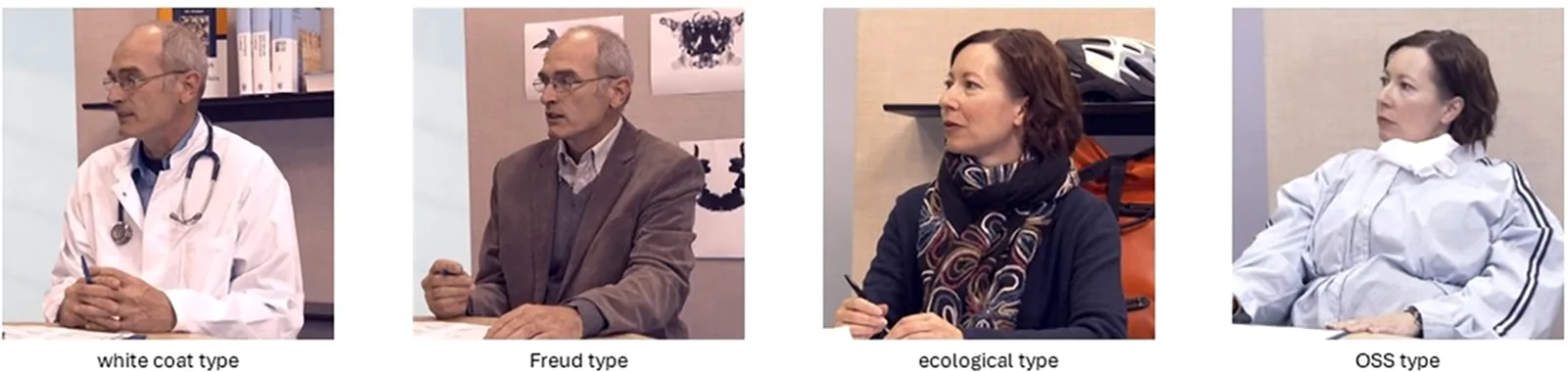
Four types of psychotherapists with four different external characteristics: the white coat type, the Freud type, the ecological type and the OSS type. The persons portrayed gave their consent for the images to be used.

Based on previous literature, we identified four relevant types: the white coat, Freud, ecological, and overweight types (25–27). These were portrayed through standardized clothing and background cues. The white coat type wore a doctor’s coat and stethoscope with medical literature in the background. The Freud type was depicted with glasses, formal clothing, and Rorschach inkblots in the background (28). The ecological type was represented by casual wool clothing, a patterned scarf, and bicycle-related items in the background (27). For the overweight type, an obesity simulation suit (grade 2 obesity; BMI, 35.0-39.9) was used to emphasize body weight. This suit increased realism and may have activated prejudice (29). See **Figure 1** for more details.

### 2.4 Measurements

Compassion, empathy, and prejudice were assessed using validated questionnaires supplemented by several self-developed items. Empathy was measured with the Jefferson Scale of Patient’s Perceptions of Physician Empathy (JSPPPE), a 5-item instrument rated on a 5-point Likert scale, with satisfactory internal consistency (Cronbach α = .58) (30). Compassion was assessed using the Physician Compassion Questionnaire, developed by Fogarty et al. and adapted by Haider et al., which measures 5 dimensions on a 10-point scale and showed high internal consistency (Cronbach α = .92) (31, 32). Prejudice was measured using the shortened semantic differential scale by Schrauth et al., comprising 18 dimensions rated on a 7-point scale, with high reliability (reliability coefficient = .85) (33, 34). In addition, we developed competence-related items based on the semantic differential scale, including 8 dimensions rated on a 10-point scale and 2 items on a 7-point Likert scale; internal consistency was high (Cronbach α = .95). Two questionnaire versions were used at different time points. Questionnaire 2 additionally included 1 item assessing the extent to which responses were influenced by the therapist’s appearance, rated on a 7-point Likert scale.

### 2.5 Procedure

**Figure 2:**
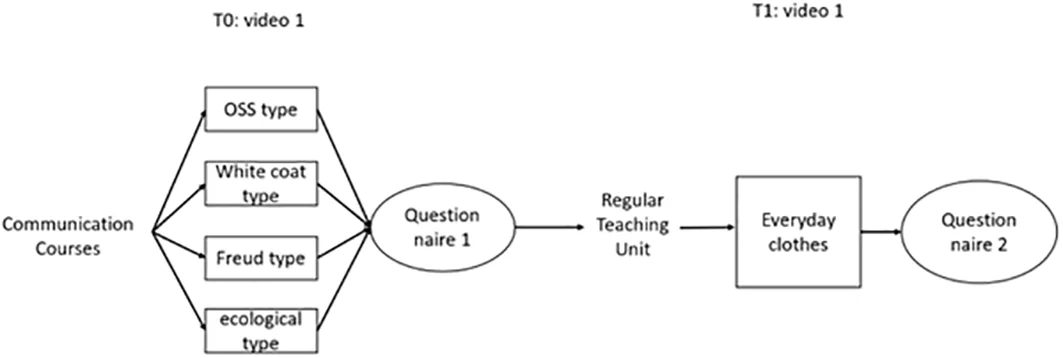
Data collection and procedure of the study.

The study was conducted within regular communication skills courses for second- and third-year medical students. Participants viewed 2 video-based simulated psychotherapy sessions. In the first session (T0), the psychotherapist was portrayed with 1 of 4 predefined external characteristics. After the routine communication skills teaching unit participants viewed a second session (T1) showing the same actor in the same setting and with the same script, but dressed in neutral everyday clothing (female actor: beige sweater; male actor: gray pullover with white stripes). Following each video, students completed Questionnaire 1 or Questionnaire 2, respectively (**Figure 2**; **Supplementary Figure A** in **Supplemental Material**). The time difference between T0 and T1 was approximately 60 minutes.

### 2.6 Data analysis

An *a priori* power analysis was conducted using G*Power with an effect size of Cohen’s (f=0.25), a significance level of (alpha =0.05), and a statistical power of 0.80, a total sample size of 180 was required for the comparison of 4 groups. Statistical analyses were performed using IBM SPSS Statistics for Windows, version 28.0.1.1 (IBM Corp). The data was tested for normal distribution by using the Kolmogorov-Smirnov-test. As the data was normally distribute we used parametric test statistics. Continuous variables are presented as mean (SD) and categorical variables as number (percentage). Missing data were handled by listwise deletion. Changes over time and differences between therapist types were examined using a mixed-design (split-plot) analysis of variance, with time (T0 vs T1) specified as a within-subject factor and therapist type as a between-subject factor. Main effects of time and therapist type as well as the time-by-type interaction were estimated. Significant omnibus tests were followed by Tukey honestly significant difference tests. A Bonferroni-adjusted significance threshold of P = .02 was applied to account for multiple comparisons. All tests were 2-sided.

## 3 Results

### 3.1 Sample size

A total of N = 330 (RR = 93.0%) out of a possible 355 students participated in the study. N = 168 of the students (50.9%) were in their third (iTüpFerl), and N = 162 (49.1%) in their sixth semester (Psychosomatic Medicine). By gender, 115 (34.8%) reported being male while 215 (65.2%) reported being female. The participants were M = 23 years old (SD = 3.35). In general, N = 80 were in the group of OSS type, N = 64 in the group of white coat type, N = 89 in the group of ecological type and N = 87 were in the group of Freud type. Please see Supplemental Material for more information.

### 3.2 Empathy

The OSS type received highest values for four (*patient’s perspective*: M = 5.51; SD = 1.07, *inquires about everyday life:* M = 5.88; SD = 1.21, *concerned about family and friends*: M = 4.46; SD = 1.40 and *understanding*: M = 5.51; SD = 1.14), followed by the ecological type, which scored higher on the item 3 whether the psychotherapist cares about the patient and their family (M = 4.34; SD = 1.41). The Freud type was considered to be the least empathetic with M = 4.37 (SD = 1.66) as an understanding psychotherapist. Significant main effects of therapist type were found for all items (p <.001), and significant time effects appeared for the following items: therapist inquires about everyday life, is concerned about family and friends and understanding. The white coat type received higher values at T0, while the ecological type, Freud type and OSS type were rated higher at T1 than at T0. The highest mean differences were found for the Freud type (d = -1.070; p <.001). *Post hoc* tests revealed significant differences primarily between the Freud type and OSS type, as well as between the Freud type and ecological type. Please see **Supplementary Table 1** in the **Supplemental Material** for more details regarding numbers and test statistics.

### 3.3 Compassion

**Figure 3:**
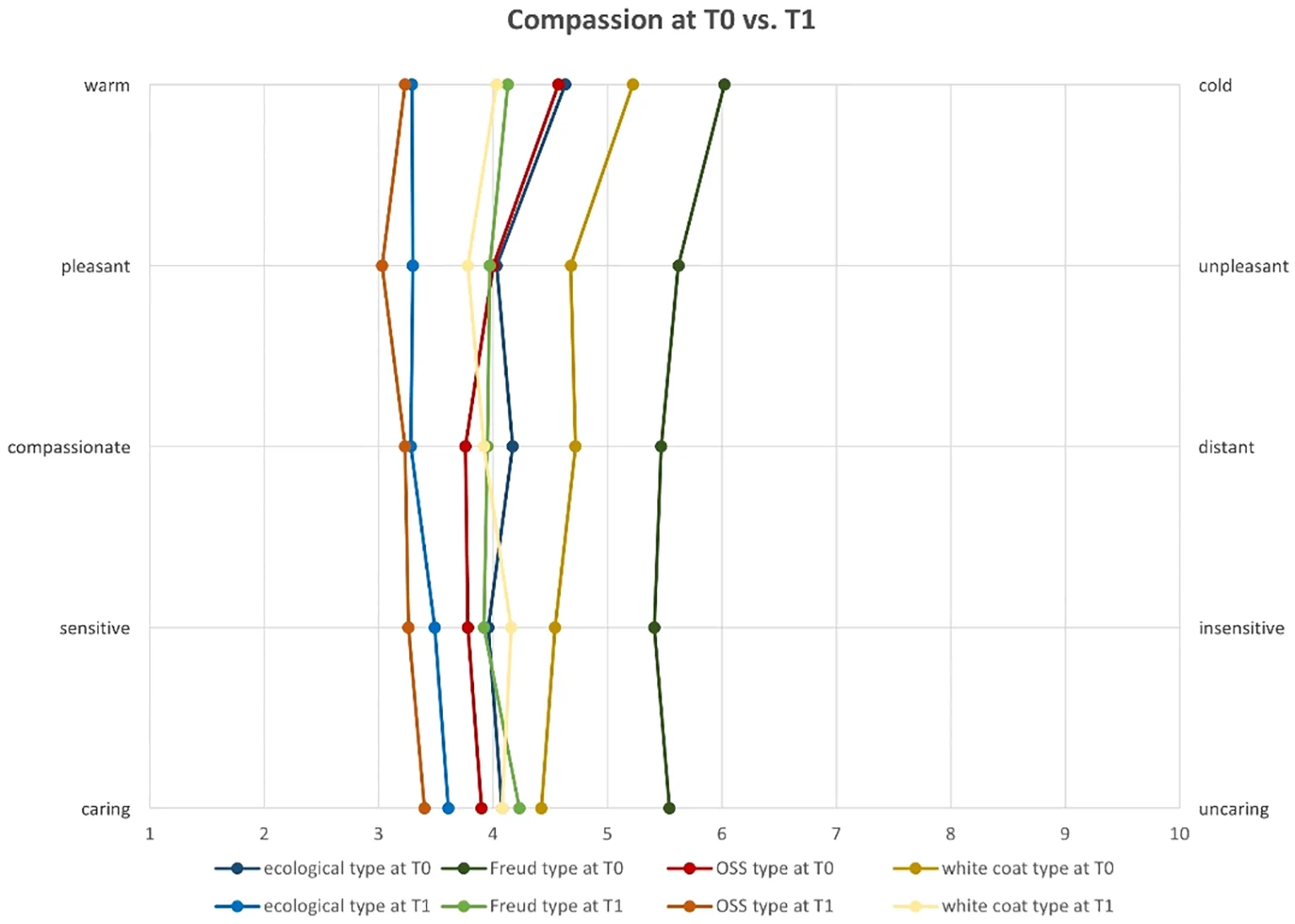
Mean values for compassion for the four types of psychotherapists at T0 and T1 on a scale from 1 to 10.

Regarding compassion, the OSS type received the highest p <.001) value with M = 3.76 (SD = 1.71), followed by the ecological type (M = 4.17; SD = 2.31). The Freud type was seen as more distant with M = 5.47 (SD = 2.29). Significant interactions between time and the types of psychotherapists were found for the items *compassionate/distant* (F (3,326) = 3,535, p = 0,015, partial η² = .032), *sensitive/insensitive* (F (3,326) = 6,039, p = <0,001, partial η² = .053) and *caring/uncaring* (F 3,326) = 4,114, p = 0,007, partial η² = .036). *Post hoc* tests showed significant differences (p <.001) between the Freud type (d = 0.940 – 1.220) and both the OSS type (d = 0.820 -1.220) and ecological type (d = 0.990 – 1.110) for all five items. Differences between the OSS type and the white coat type surfaced for *compassionate/distant* (d = -0.820) and *sensitive/insensitive* (d = -0-830). In general, mean values at T0 were higher than at T1. The greatest mean differences again appeared for the Freud type. **Figure 3** presents the mean values for compassion for the four different types of psychotherapists at T0 and T1. **Supplementary Table 2** in the **Supplemental Material** shows the numbers and test statistic in more details.

### 3.4 Prejudice

**Figure 4:**
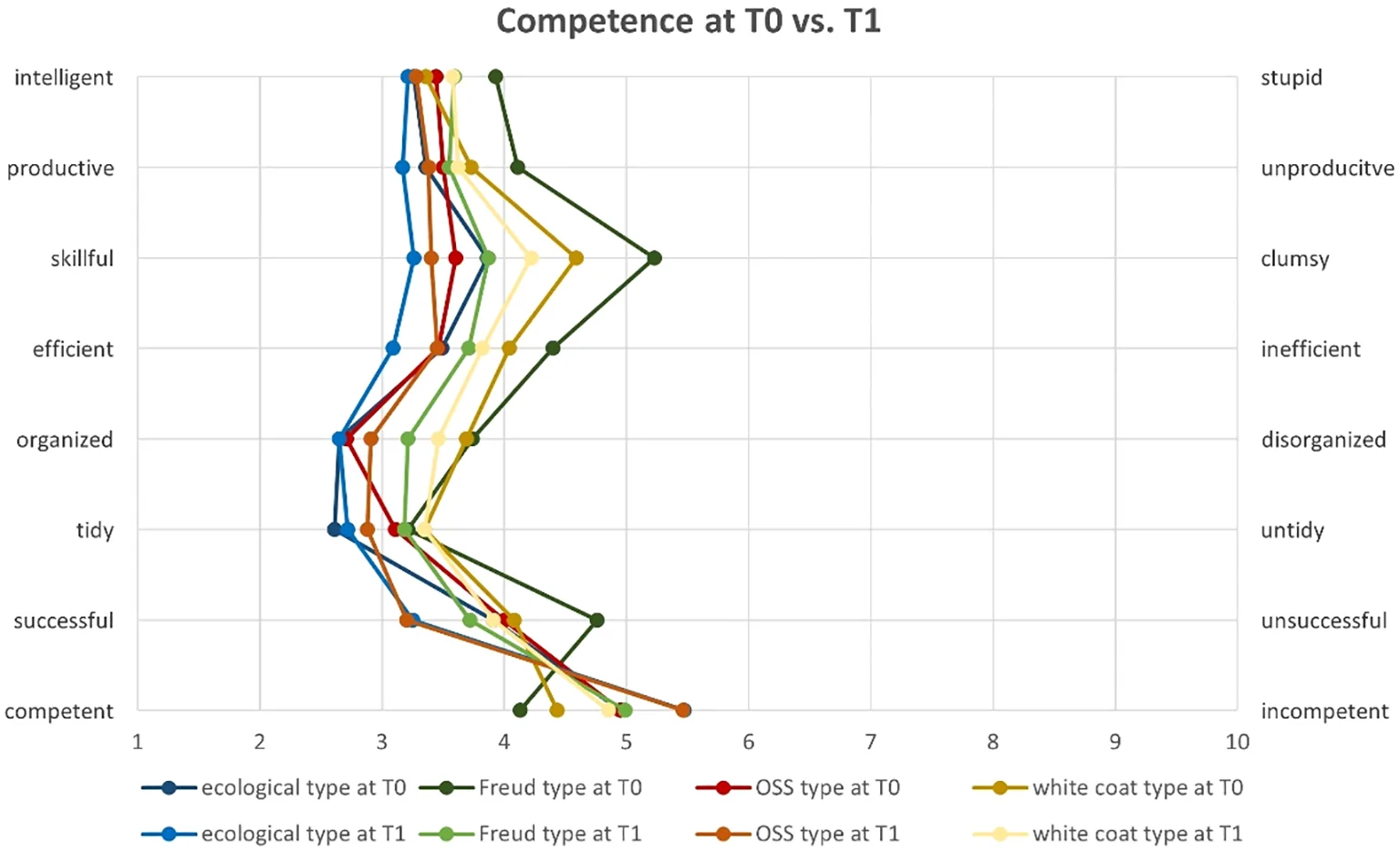
Mean values for prejudice for the four types at T0 and T13.5 Competence.

As shown in **Figure 4**, the four therapist types differed systematically. Overall, the Freud type received the least favorable ratings across most items (means ranging from M = 3.56, SD = 1.40 to M = 5.46, SD = 1.36), followed by the white coat type (means ranging from M = 3.77, SD = 0.87 to M = 4.84, SD = 1.55). In contrast, the OSS type was rated most favorably on several interpersonal characteristics and was perceived as the most *realistic* (M = 3.34, SD = 1.45), *active* (M = 5.24, SD = 1.13), *likable* (M = 4.81, SD = 1.32), *trustworthy* (M = 5.34, SD = 1.12), and *confident* (M = 5.33, SD = 1.06). The ecological type was rated as the most *progressive* (M = 4.80, SD = 1.58), *selfless* (M = 4.57, SD = 1.07), *poor* (M = 3.91, SD = 0.74), *reliable* (M = 5.37, SD = 1.11), and *democratic* (M = 4.89, SD = 1.50), but also received the second-highest rating on *superficial* (M = 4.45, SD = 1.55). The OSS type received the second-lowest ratings on *rich/poor* (M = 3.71, SD = 0.81) and *authoritarian/democratic* (M = 4.61, SD = 1.48). **Figure 4** presents these differences regarding prejudice for the four types of psychotherapists at T0 and T1.

There were significant type × time interactions for *insecure*, F (3, 326) = 4.67, p = .003, partial η² = .041, and *rough*, F (3, 324) = 5.05, p = .002, partial η² = .045. Significant main effects of time were observed for nearly all items (p <.01). Across almost all items, ratings were higher at T1 than at T0. The Freud type showed the largest mean differences over time in 10 of the 18 items. *Post hoc* tests revealed numerous significant differences, particularly between the Freud type and both the OSS and ecological types, as well as between the white coat type and the other types. **Supplementary Table 3** in the **Supplemental Material** shows more numbers and test statistics in detail.

**Figure 5:**
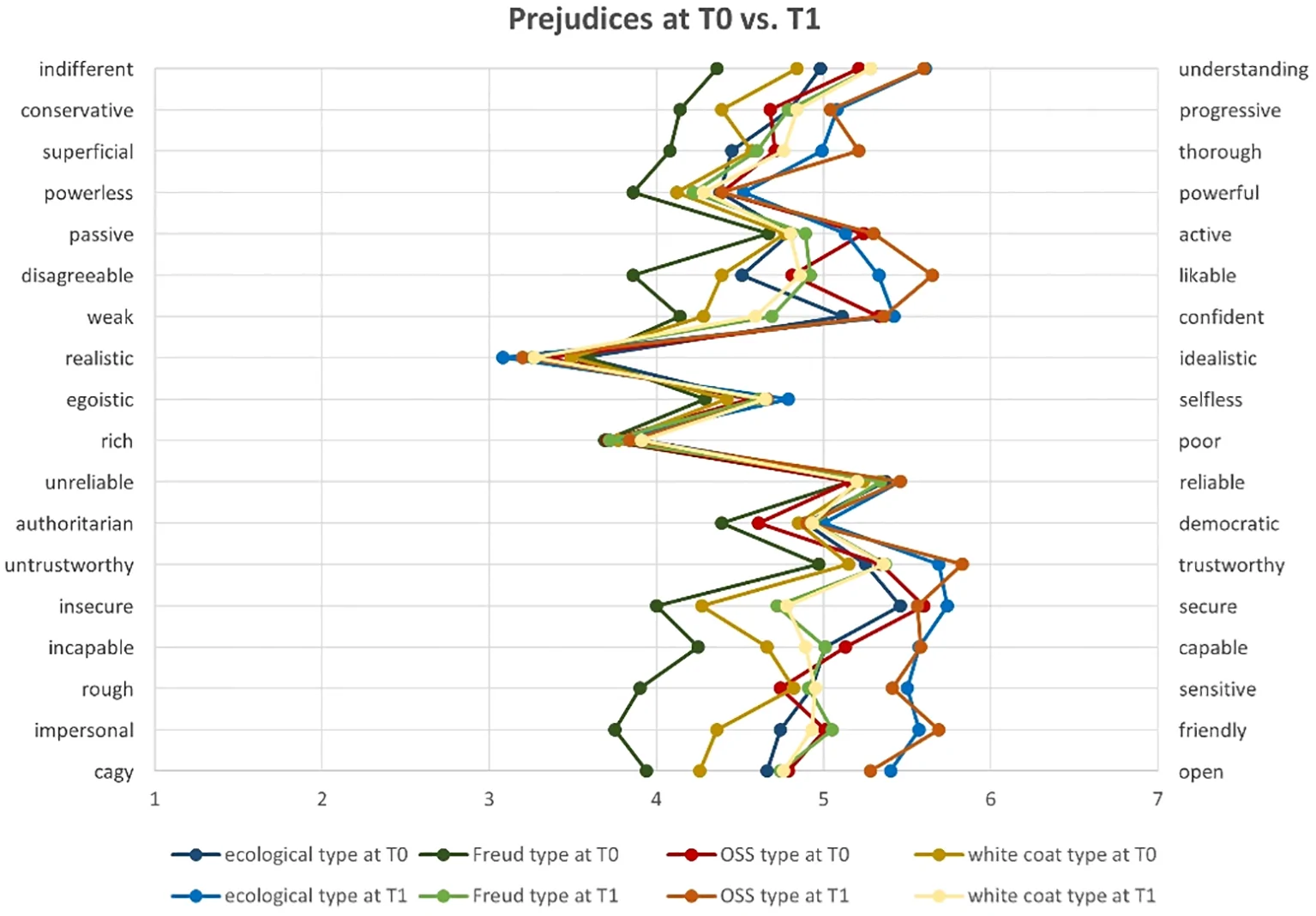
Mean values for competence for the four types of psychotherapists at T0 and T1.

The Freud type was rated as *unsuccessful* (M = 4.76; SD = 2.15), *inefficient* (M = 4.40; SD = 2.34) and *clumsy* (M = 5.23; SD = 2.17). The OSS type (M = 4.95; SD = 1.22) and ecological type (M = 4.95, SD = 1.36) were rated as comparably highly *competent* and the Freud type as being the least (M = 4.13; SD = 1.49). The students stated that they would be most likely to seek treatment from the OSS type (M = 3.81; SD = 1.86). In contrast, the Freud type had the lowest rating when asked for treatment (M = 2.89; SD = 1.72). **Figure 5** shows the different mean values for each type of psychotherapist at T0 and T1.

A significant interaction between the time and type emerged only for *skillful/clumsy* (F (3,324) = 6,127, p = <0,001, partial η² = .054). For both time points, statistically significant main effects (p <.02) emerged for six of the nine items. The main effects for the types can be calculated for seven of the nine items. For nearly all items, the mean values at T0 were higher than at T1. The Freud type again showed the largest mean differences on most items. *Post hoc* comparisons revealed significant differences between the Freud type and the ecological type or OSS type, as well as between the ecological type and white coat type or the OSS and white coat type. **Supplementary Table 4** in the **Supplemental Material** provide details concerning the abovementioned results.

### 3.6 Influence of psychotherapist’s appearance on students’ responses

When asked whether the psychotherapists’ appearance influenced their responses, students gave the lowest scores to the ecological type (M = 3.43 ± 1.65) and the highest to the OSS type (M = 4.00 ± 1.86). However, the result was not statistically significant (p = .167). In general, students agreed that the psychotherapist’s appearance influenced their responses (Freud type: M = 3.63 ± 1.55; white coat: M = 3.81 ± 1.77).

## 4 Discussion

In this longitudinal observational study, we examined whether psychotherapists’ therapist type influenced medical students’ evaluations of competence, empathy, and appearance-based bias.

### 4.1 Principal findings

Across measures of empathy, compassion, competence, and attributed traits, the OSS type received the most favorable ratings, followed by the ecological type, whereas the Freud type was rated least favorably. Psychotherapists shown in everyday clothing were generally evaluated more positively than those shown in stylized roles, particularly the Freud type. Students also indicated that, if they needed psychotherapy themselves, they would be most likely to seek treatment from the OSS type. Although self-reported influence of appearance was not statistically significant, students most often reported being influenced by the OSS type. Only compassion showed a significant time-by-type interaction, suggesting that some evaluative dimensions may change over time depending on external characteristics.

### 4.2 Interpretation and relation to prior work

Our findings add nuance to previous research on first impressions and clinical attire. Prior studies have shown that white coats may convey competence, trustworthiness, and scientific credibility, but can also create distance and hinder patient-centered communication (4, 9, 10, 14, 16). In our sample, however, the white-coat type was evaluated less favorably than expected. One possible explanation is the psychotherapeutic context, in which warmth, openness, and nonverbal attunement may be valued more than signals of authority, making formal medical attire appear distancing (10, 16).

Most notably, the OSS type received the highest ratings across several domains, including competence-related and positively valenced traits. This contrasts with prior research on weight-related stigma and professional disadvantage among physicians with overweight (11, 17–21). At the same time, it is consistent with evidence suggesting that weight bias may be less pronounced when judging a physician’s ability to listen to and understand patients (13). It is also possible that students responded less to body weight itself than to another salient visual cue, producing a halo effect (11).

The consistently low ratings of the Freud type, together with the more favorable evaluations of everyday clothing, suggest that appearance cues can shape judgments even when clinical content is held constant. This is consistent with theories of impression formation in which salient visual features influence global evaluations (7), and negative cues may weigh more heavily than positive ones (35).

### 4.3 Role of nonverbal behavior and potential confounding

An important consideration is that the OSS type and ecological type were portrayed by the same actress, and these portrayals were generally evaluated more positively. Students noted during the post-session discussion that—despite the same script—the actors’ nonverbal behavior (eg, body language, gestures, facial expressions) appeared different and influenced their judgments. This raises the possibility that observed differences reflect not only external characteristics (attire/body weight) but also actor-specific nonverbal cues. One reason might be that the OSS type and ecological type were portrayed by a woman as gender and a person’s attire might affect the students (36). Future studies should more tightly standardize nonverbal behavior and context variables (e.g., using the same actor across conditions, counterbalancing actors across appearance types, or using controlled photographic stimuli).

### 4.4 Implications for medical education and practice

From an educational perspective, these findings support integrating explicit reflection on appearance-based bias into communication training. Even subtle visual cues may influence perceptions of empathy and competence - two attributes central to therapeutic success - and may affect whom patients choose to consult. Students should therefore be encouraged to reflect on the impressions their appearance may create. Teaching formats could include guided debriefing of first impressions, structured reflection on implicit assumptions, and training to distinguish interpersonal judgments from appearance-based heuristics. For clinicians and psychotherapists, the findings suggest that external presentation may shape how professional qualities are perceived, particularly in relational disciplines.

### 4.5 Limitations

Several limitations merit attention. First, actor-related effects and subtle differences in nonverbal behavior may have influenced students’ ratings, limiting causal attribution to appearance characteristics alone. Although the script and setting were standardized and the same actors were used across conditions, complete control of nonverbal cues is not feasible. Even minor variations may have affected perceptions independently of the manipulated visual features, introducing potential residual confounding. Second, the operationalization of types (e.g., OSS) may have combined multiple cues (body weight, dress style, grooming), complicating interpretation. Future studies should use fully counterbalanced designs (e.g., multiple actors per condition or the same actor across conditions) and more tightly controlled stimuli to separate appearance cues from nonverbal delivery. Moreover, we would like to raise awareness that the videos may not fully replicate real clinical encounters, so the ecological validity is limited. Furthermore, the results should not be generalized and future studies should explore cultural context in more detail. Finally, we had to use the Jefferson Scale of Patient’s Perceptions of Physician Empathy as the observer scale had not been published yet. In this way, however, the students were able to consider both perspectives—the patient’s perspective and how this influences their appearance as future doctors. In future studies, we would use the observer scale.

## 5 Conclusion

In this longitudinal observational study, medical students’ evaluations of psychotherapists varied by external presentation, including both stylized appearance types and everyday attire. These findings suggest that appearance-based cues can shape perceptions of empathy and competence—key determinants of therapeutic alliance and clinical decision-making—highlighting the importance of addressing appearance-related bias in undergraduate medical training. Creating structured opportunities for reflection may help students recognize and manage implicit assumptions in clinical communication and support more equitable, objective care.

## Data Availability

The raw data supporting the conclusions of this article will be made available by the authors, without undue reservation.
