# Supplementary material for: Perceived empathy and competence across psychotherapist appearance types: a quasi-experimental repeated-measures study
Source: Front Psychiatry. 2026 Aug 13;17:1896992. doi: 10.3389/fpsyt.2026.1896992 (PMC13518353; doi:10.3389/fpsyt.2026.1896992)
Supplement: Supplementary file 1 [file Table1.docx]

Appendix

Figure A: Potential and actual study population, the distribution of students between the two courses iTüpFerl and Psychosomatic Medicine (i.e. indicated by the number and percentage of the actual study population) and the four types of psychotherapists

Table 1: Results for the Jefferson Scale of Patient’s Perceptions of Physician Empathy (JSPPPE), used to measure physicians’ empathy as perceived by their patients at T0 vs. T1.

| **Item** | **Ecological type (eco)** | | **Freud type (Freud)** | | **Obesity simulation suit type (OSS)** | | **White coat type (coat)** | | **Main effects** | | **Post hoc** | | |
| --- | --- | --- | --- | --- | --- | --- | --- | --- | --- | --- | --- | --- | --- |
| The therapist | *M*_T0_ | *M*_T1_ | *M*_T0_ | *M*_T1_ | *M*_T0_ | *M*_T1_ | *M*_T0_ | *M*_T1_ | Time point*: p* | Type*: p* | Tukey’s HSD | Difference | *p* |
| 1) looks from patient’s perspective | 5.34 ± 1.21 | 5.43 ± 1.13 | 4.37 ± 1.37 | 4.70 ± 1.37 | 5.45 ± 0.99 | 5.51 ± 1.07 | 4.92 ± 1.52 | 4.78 ± 1.56 | .256 | <.001 | eco – Freud eco – coat Freud – OSS  OSS – coat | 0.850  0.530  -0.950  0.630 | <.001  .011  <.001  .002 |
| 2) inquires about everyday life | 6.19 ± 1.20 | 5.97 ± 1.20 | 5.83 ± 1.35 | 5.37 ± 1.52 | 6.29 ± 0.93 | 5.88 ± 1.21 | 5.85 ± 1.43 | 5.24 ± 1.65 | <.001 | .001 | eco – coat | 0.530 | .018 |
| 3) is concerned about patient and family | 4.28 ± 1.53 | 4.34 ± 1.41 | 3.36 ± 1.41 | 3.97 ± 1.37 | 4.00 ± 1.26 | 4.46 ± 1.40 | 3.65 ± 1.53 | 3.88 ± 1.36 | <.001 | <.001 | eco – Freud  Freud – OSS | 0.650  -0.570 | .002  .014 |
| 4) understands emotions, feelings and worries | 5.08 ± 1.46 | 5.48 ± 1.16 | 4.18 ± 1.54 | 4.70 ± 1.37 | 5.31 ± 1.13 | 5.51 ± 1.14 | 4.73 ± 1.48 | 4.65 ± 1.42 | .001 | <.001 | eco – Freud  eco – coat  Freud – OSS  OSS – coat | 0.840  0.590  -0.970  0.720 | <.001  .005  <.001  <.001 |
| 5) is an understanding psychotherapist | 5.35 ± 1.43 | 5.47 ± 1.31 | 4.37 ± 1.66 | 4.78 ± 1.43 | 5.59 ± 1.25 | 5.70 ± 1.27 | 5.03 ± 1.56 | 4.77 ± 1.51 | .257 | <.001 | eco – Freud  Freud – OSS  OSS – coat | 0.830  -1.070  0.750 | <.001  <.001  <.001 |

Table 2: Results for the Physician compassion questionnaire used to measure physicians’ compassion as perceived by patients

| **Item** | **Ecological type (eco)** | | **Freud type (Freud)** | | **Obesity simulation suit type (OSS)** | | **White coat type (coat)** | | **Main effects** | | **Post hoc** | | |
| --- | --- | --- | --- | --- | --- | --- | --- | --- | --- | --- | --- | --- | --- |
|  | *M*_T0_ | *M*_T1_ | *M*_T0_ | *M*_T1_ | *M*_T0_ | *M*_T1_ | *M*_T0_ | *M*_T1_ | Time point*: p* | Type*: p* | Tukey’s HSD | Difference | *p* |
| warm / cold | 4.63 ± 2.50 | 3.29 ± 1.88 | 6.02 ± 2.07 | 4.13 ± 1.87 | 4.57 ± 2.13 | 3.23 ± 1.81 | 5.22 ± 2.16 | 4.03 ± 1.66 | <.001 | <.001 | eco – Freud Freud – OSS | -1.110  1.170 | <.001  <.001 |
| pleasant / unpleasant | 4.03 ± 2.22 | 3.30 ± 1.96 | 5.62 ± 2.27 | 3.97 ± 1.70 | 4.00 ± 1.95 | 3.03 ± 1.72 | 4.68 ± 2.42 | 3.78 ± 1.72 | <.001 | <.001 | eco – Freud  Freud – OSS | -1.120  1.290 | <.001  <.001 |
| compassionate / distant | 4.17 ± 2.31 | 3.28 ± 1.60 | 5.47 ± 2.29 | 3.95 ± 1.56 | 3.76 ± 1.71 | 3.23 ± 1.49 | 4.72 ± 2.29 | 3.92 ± 1.69 | <.001 | <.001 | eco – Freud  Freud – OSS  OSS – coat | -0.990  1.220  -0.820 | <.001 <.001  .009 |
| sensitive / insensitive | 3.96 ± 2.06 | 3.49 ± 1.69 | 5.41 ± 2.05 | 3.92 ± 1.59 | 3.78 ± 1.83 | 3.26 ± 1.60 | 4.54 ± 1.95 | 4.16 ± 1.66 | <.001 | <.001 | eco – Freud  Freud – OSS  OSS – coat | -0.940  1.150  -0.830 | <.001  <.001  .004 |
| caring / uncaring | 4.08 ± 2.07 | 3.61 ± 1.74 | 5.54 ± 2.26 | 4.23 ± 1.58 | 3.90 ± 1.69 | 3.40 ± 1.67 | 4.42 ± 2.16 | 4.08 ± 1.65 | <.001 | <.001 | eco – Freud  Freud – OSS | -1.040  1.240 | <.001  <.001 |

Table 3: Results on the semantic differential scale used to quantify students’ prejudices

| **Item** | **Ecological type (eco)** | | **Freud type (Freud)** | | **Obesity simulation suit type (OSS)** | | **White coat type (coat)** | | **Main effects** | | **Post hoc** | | |
| --- | --- | --- | --- | --- | --- | --- | --- | --- | --- | --- | --- | --- | --- |
|  | *M*_T0_ | *M*_T1_ | *M*_T0_ | *M*_T1_ | *M*_T0_ | *M*_T1_ | *M*_T0_ | *M*_T1_ | *p* (for time) | *p* (for type) | Tukey’s HSD | Difference | *p* |
| Indifferent / understanding | 4.98 ± 1.68 | 5.61 ± 1.27 | 4.36 ± 1.46 | 5.28 ± 1.09 | 5.21 ± 1.33 | 5.60 ± 1.21 | 4.84 ± 1.55 | 5.28 ± 1.12 | <.001 | .004 | Freud – OSS | -0.590 | .005 |
| Conservative / progressive | 4.80 ± 1.58 | 5.08 ± 1.27 | 4.14 ± 1.64 | 4.79 ± 1.33 | 4.68 ± 1.44 | 5.04 ± 1.11 | 4.39 ± 1.81 | 4.84 ± 1.49 | <.001 | .078 |  |  |  |
| Superficial / thorough | 4.45 ± 1.55 | 4.99 ± 1.42 | 4.08 ± 1.48 | 4.60 ± 1.38 | 4.71 ± 1.30 | 5.21 ± 1.27 | 4.57 ± 1.53 | 4.76 ± 1.30 | <.001 | .013 | Freud – OSS | -0.620 | .007 |
| Powerless / powerful | 4.38 ± 0.87 | 4.52 ± 0.93 | 3.86 ± 1.05 | 4.22 ± 0.83 | 4.39 ± 0.97 | 4.39 ± 1.00 | 4.12 ± 0.94 | 4.28 ± 1.04 | .003 | .005 | eco – Freud | 0.410 | .006 |
| Passive / active | 4.80 ± 1.26 | 5.13 ± 1.23 | 4.67 ± 1.47 | 4.89 ± 1.15 | 5.24 ± 1.13 | 5.30 ± 1.08 | 4.77 ± 1.32 | 4.80 ± 1.07 | .017 | .011 | Freud – OSS | -0.490 | 0.015 |
| Disagreeable / likable | 4.51 ± 1.74 | 5.33 ± 1.36 | 3.86 ± 1.54 | 4.92 ± 1.24 | 4.81 ± 1.32 | 5.65 ± 1.22 | 4.39 ± 1.58 | 4.86 ± 1.43 | <.001 | <.001 | Freud – OSS OSS – coat | -0.840 0.600 | <.001  .013 |
| Weak /  confident | 5.11 ± 1.42 | 5.42 ± 1.28 | 4.14 ± 1.70 | 4.69 ± 1.28 | 5.33 ± 1.06 | 5.36 ± 1.09 | 4.28 ± 1.68 | 4.59 ± 1.34 | <.001 | <.001 | eco – Freud eco – coat  Freud – OSS  OSS – coat | 0.850  0.820  -0.930  0.900 | <.001  <.001  <.001  <.001 |
| Realistic / idealistic | 3.56 ± 1.40 | 3.08 ± 1.13 | 3.59 ± 1.51 | 3.26 ± 1.04 | 3.34 ± 1.45 | 3.20 ± 1.26 | 3.49 ± 1.29 | 3.27 ± 1.09 | <.001 | .799 |  |  |  |
| Egoistic / selfless | 4.57 ± 1.07 | 4.79 ± 0.83 | 4.29 ± 0.97 | 4.61 ± 0.80 | 4.56 ± 0.82 | 4.66 ± 0.84 | 4.42 ± 0.82 | 3.91 ± 0.97 | <.001 | .206 |  |  |  |
| Rich / poor | 3.91 ± 0.74 | 3.72 ± 0.64 | 3.69 ± 0.82 | 3.72 ± 0.72 | 3.71 ± 0.81 | 3.84 ± 0.79 | 3.77 ± 0.87 | 3.91 ± 0.97 | .650 | .602 |  |  |  |
| Unreliable / reliable | 5.37 ± 1.11 | 5.46 ± 1.00 | 5.16 ± 1.21 | 5.34 ± 1.01 | 5.16 ± 1.15 | 5.46 ± 1.02 | 5.24 ± 1.17 | 5.20 ± 1.17 | .029 | .581 |  |  |  |
| Authoritarian / democratic | 4.89 ± 1.50 | 5.00 ± 1.19 | 4.39 ± 1.57 | 4.94 ± 1.29 | 4.61 ± 1.48 | 4.90 ± 1.12 | 4.85 ± 1.54 | 4.93 ± 1.16 | .002 | .379 |  |  |  |
| Untrustworthy / trustworthy | 5.25 ± 1.32 | 5.69 ± 1.22 | 4.97 ± 1.58 | 5.37 ± 1.19 | 5.34 ± 1.12 | 5.83 ± 0.93 | 5.15 ± 1.51 | 5.36 ± 1.33 | <.001 | .078 |  |  |  |
| Insecure / secure | 5.46 ± 1.36 | 5.74 ± 1.13 | 4.00 ± 1.81 | 4.72 ± 1.43 | 5.60 ± 0.92 | 5.56 ± 0.88 | 4.27 ± 1.73 | 4.78 ± 1.50 | <.001 | <.001 | eco – Freud eco – coat  Freud – OSS  OSS – coat | 1.240  1.070  -1.220  1.050 | <.001  <.001  <.001  <.001 |
| Incapable / capable | 5.01 ± 1.42 | 5.57 ± 1.18 | 4.25 ± 1.55 | 5.01 ± 1.24 | 5.13 ± 1.18 | 5.58 ± 1.12 | 4.66 ± 1.55 | 4.89 ± 1.28 | <.001 | <.001 | eco – Freud  Freud – OSS  OSS – coat | 0.660  -0.720  0.570 | .001  <.001  .014 |
| rough / sensitive | 4.93 ± 1.40 | 5.50 ± 1.15 | 3.90 ± 1.39 | 4.91 ± 1.04 | 4.74 ± 1.41 | 5.41 ± 1.06 | 4.82 ± 1.31 | 4.95 ± 1.27 | <.001 | <.001 | eco – Freud  Freud – OSS  Freud – coat | 0.810  -0.660  -0.480 | <.001  <.001  .017 |
| Impersonal / friendly | 4.74 ± 1.80 | 5.57 ± 1.51 | 3.75 ± 1.90 | 5.05± 1.39 | 5.01 ± 1.64 | 5.69 ± 1.29 | 4.36 ± 1.83 | 4.93 ± 1.61 | <.001 | <.001 | eco – Freud  Freud – OSS  OSS – coat | 0.760  -0.950  0.700 | .001  <.001  .008 |
| Cagy / open | 4.66 ± 1.49 | 5.40 ± 1.22 | 3.94 ± 1.50 | 4.74 ± 1.36 | 4.79 ± 1.55 | 5.28 ± 1.16 | 4.26 ± 1.64 | 4.76 ± 1.27 | <.001 | <.001 | eco – Freud  Freud – OSS | 0.690  -0.690 | .001  .001 |

Table 4: Results on the semantic differential scale used to quantify competence

| **Item** | **Ecological type (eco)** | | **Freud type (Freud)** | | **Obesity simulation suit type (OSS)** | | **White coat (coat)** | | **Main effects** | | **Post hoc** | | |
| --- | --- | --- | --- | --- | --- | --- | --- | --- | --- | --- | --- | --- | --- |
|  | *M*_T0_ | *M*_T1_ | *M*_T0_ | *M*_T1_ | *M*_T0_ | *M*_T1_ | *M*_T0_ | *M*_T1_ | *p* (for time) | *p* (for type) | Tukey’s HSD | Difference | *p* |
| Intelligent / stupid | 3.26 ± 1.78 | 3.21 ± 1.66 | 3.93 ± 1.87 | 3.59 ± 1.52 | 3.44 ± 1.52 | 3.28 ± 1.69 | 3.36 ± 1.79 | 3.58 ± 1.73 | .282 | .142 |  |  |  |
| Productive / unproductive | 3.36 ± 2.01 | 3.17 ± 1.76 | 4.11 ± 2.25 | 3.55 ± 1.58 | 3.50 ± 1.81 | 3.38 ± 1.57 | 3.73 ± 1.89 | 3.62 ± 1.74 | .011 | .101 |  |  |  |
| Skilful / clumsy | 3.86 ± 2.02 | 3.26 ± 1.66 | 5.23 ± 2.17 | 3.87 ± 1.85 | 3.60 ± 1.67 | 3.40 ± 1.61 | 4.59 ± 2.20 | 4.22 ± 2.04 | <.001 | <.001 | eco – Freud  eco – coat  Freud – OSS  OSS – coat | -0.980  -0.830  1.050  -0.910 | <.001  .009  <.001  .005 |
| Efficient / inefficient | 3.49 ± 2.26 | 3.09 ± 1.83 | 4.40 ± 2.34 | 3.71 ± 1.78 | 3.46 ± 1.85 | 3.45 ± 1.86 | 4.04 ± 2.10 | 3.82 ± 1.90 | .002 | .015 |  |  |  |
| Organised / disorganised | 2.65 ± 1.76 | 2.65 ± 1.46 | 3.74 ± 2.09 | 3.21 ± 1.49 | 2.71 ± 1.41 | 2.91 ± 1.53 | 3.69 ± 2.33 | 3.46 ± 1.81 | .159 | <.001 | eco – Freud  eco – coat OSS – coat | -0.820 -0.920  -0.760 | .002  <.001  .010 |
| Tidy / untidy | 2.61 ± 1.44 | 2.72 ± 1.37 | 3.21 ± 1.63 | 3.18 ± 1.39 | 3.11 ± 1.75 | 2.88 ± 1.70 | 3.35 ± 1.84 | 3.35 ± 1.76 | .705 | .007 | eco – coat | -0.690 | .006 |
| Successful / unsuccessful | 3.91 ± 1.91 | 3.25 ± 1.79 | 4.76 ± 2.15 | 3.72 ± 1.63 | 4.00 ± 1.79 | 3.20 ± 1.49 | 4.08 ± 1.96 | 3.91 ± 1.60 | <.001 | .017 |  |  |  |
| Competent / incompetent | 4.95 ± 1.36 | 5.47 ± 1.31 | 4.13 ± 1.49 | 4.99 ± 1.25 | 4.95 ± 1.22 | 5.46 ± 1.30 | 4.43 ± 1.55 | 4.85 ± 1.42 | <.001 | <.001 | eco – Freud  eco – coat  Freud – OSS | 0.650  0.570  -0.650 | .002  .016  .003 |
| Own treatment | 3.63 ± 1.99 | 4.69 ± 1.82 | 2.89 ± 1.72 | 4.06 ± 1.67 | 3.81 ± 1.86 | 4.90 ± 1.68 | 3.32 ± 1.95 | 3.95 ± 1.83 | <.001 | .001 | Freud – OSS | -0.880 | .003 |
